# Impact of Urbanization on Ecosystem Services Balance in the Han River Ecological Economic Belt, China: A Multi-Scale Perspective

**DOI:** 10.3390/ijerph192114304

**Published:** 2022-11-01

**Authors:** Weisong Li, Wanxu Chen, Jiaojiao Bian, Jun Xian, Li Zhan

**Affiliations:** 1 Hubei University of Economics, Wuhan 430205, China; 2 Collaborative Innovation Center for Emissions Trading System Co-Constructed by the Province and Ministry, Wuhan 430205, China; 3 School of Geography and Information Engineering, China University of Geosciences (Wuhan), Wuhan 430074, China; 4 Experimental Teaching Centre, Hubei University of Economics, Wuhan 430205, China; 5 School of Tourism and Hospitality Management, Hubei University of Economics, Wuhan 430205, China

**Keywords:** ecosystem services balance, urbanization level, geographically weighted regression model, multi-scale analysis, Han River ecological economic belt, China

## Abstract

Urbanization intensification seriously interferes with the supply capacity and demand level of ecosystem services (ESs); therefore, it affects the balance state of ESs. Coordination of urbanization development and ecosystem protection in the ecological economic belt is vital for ecological protection and high-quality development of the ecological economic belt. However, previous studies lacked multi-scale analysis of the impact of urbanization elements on the ESs balance index (ESBI) in the ecological economic belt. In this study, a geographically weighted regression model was employed to measure the spatial non-stationary patterns associated with the impact of urbanization elements on the ESBI at 5 km and 10 km in the Han River Ecological Economic Belt (HREEB) in China based on land use data. The main findings were shown as follows. The supply capacity and demand level of ESs in the HREEB increased from 2000 to 2020 simultaneously, while the ESBI showed a decreasing trend. In mountainous areas, the ESBIs were evidently higher than those in the plain areas. During the study period, the urbanization level in the HREEB improved evidently, and the urbanization levels of the middle and lower reaches of the Hanjiang River were relatively high. Significant spatial dependence between urbanization elements and the ESBI was identified. Urbanization had significant positive and negative impacts on ESBI, and there were significant differences among different scales. The findings of this study can act as a decision-making reference for ecological protection and high-quality development of the HREEB and can also provide a perspective for exploring the impact of urbanization on the ESBI of the ecological economic belt in other similar regions.

## 1. Introduction

Urbanization, an objective trend of human social development and an important symbol of national modernization, is a natural, historical process accompanied by landscape change, non-agricultural industry, and a non-agricultural population [1,2]. Urbanization promotion interferes with the supply capacity of ecosystem services (ESs) to a certain extent and promotes an increase in the demand for ESs, which leads to an imbalance between supply and demand (S&D) for ESs and causes severe ecological issues [3,4,5,6,7]. The over-dependence on certain services provided by the ecosystem during rapid urbanization aggravates the trade-offs between such services and the supply of other ESs, resulting in changes to the supply of other ESs [8,9,10]. The unbalanced demand for ESs causes a vicious cycle of socioeconomic and environmental injustice, which leads to trade-offs between the demand for ESs and the difference between the S&D of ESs [11,12,13,14,15]. However, few previous studies have analyzed the effect of multi-scale spatially varying relationships between urbanization elements on the ESs balance. Therefore, it is vital to explore the impact of urbanization elements on the ESs balance.

ESs and human well-being are closely linked [16,17,18]. There has been a wealth of research on ESs [5,12,19,20]. Existing studies have reported extensively on ESs assessments, influencing factors, trade-offs and synergies, the S&D balance, and scenario predictions [13,21,22,23,24,25]. Revealing the formation mechanism, expression form, and basic characteristics of ESs S&D can provide scientific guidance for reducing the trade-offs between the S&D of ESs [13,26,27,28]. The research contents of ESs S&D mainly include the spatial identification of S&D, the assessment of quality and quantity, the dynamic impact mechanism, and the equilibrium and spatial matching analysis [29,30,31,32]. Quantitative assessment of the S&D of ESs is currently a major difficulty. S&D for ESs can be measured using a variety of models, such as models based on ecological processes, the spatial mapping method based on remote sensing and geographic information system technology, the matrix method based on expert knowledge, and the questionnaire survey method [12,13,20,27]. For example, Wang et al. (2019) evaluated the ESs S&D index through the ESs provision index and the land development index [4]. Zhang et al. (2021) used the InVEST model to measure the S&D of ESs in the Pearl River Delta of China [28]. Chen et al. (2020) evaluated the ESs balance index (ESBI) based on a revised ESs matrix method [33]. Based on different definitions and requirements, different models have been used to measure the S&D index of ESs [21,28,33]. The ESs matrix method proposed by Burkhard et al. (2012) provided a new perspective for ESs S&D [34]. This method has low data requirements, simple operation, and highly feasible evaluation results [21,33,35,36]. Therefore, this study will use the ESs matrix method to measure the ESs supply, demand, and balance. Although existing literature has emphasized assessments of ES S&D, these studies did not sufficiently explore the spatial dependence of ES balance and its response to urbanization [28,37,38,39]. In addition, previous studies have been conducted on macro scales, such as the national scale, provincial scale, and prefecture-level city scale [21,30,35,40]; there is a lack of studies on the S&D of ESs on the multiple scales. 

Urbanization is a long-term historical process that is accompanied by economic urbanization, social urbanization, spatial urbanization, and population urbanization [1,38,41]. According to the United Nations 2018 World Urbanization Trend (https://population.un.org/wup/DataQuery/ accessed on 1 January 2022), the global urbanization rate has risen from 24% in 1950 to 55% in 2018 and is expected to reach 68% by 2050. On the one hand, rapid urbanization will intensify the disturbance to the ecosystem [5,28,30,42]. Specifically, during the process of urbanization, the non-agricultural population, non-agricultural industry, and urban space expansion will inevitably intensify the disturbance to the ecosystem, leading to ecological problems, such as ecosystem degradation, habitat quality reduction, and biodiversity reduction [43,44,45]. For example, as the carrier of urbanization, the rapid expansion of urban land leads to the occupation of a large amount of cultivated land and ecological land, which inevitably reduces the supply services and regulating services. On the other hand, urbanization will promote an increase in the demand for ESs [3,4]. Urbanization is also a process in which the population, economy, and other factors are concentrated in urban areas, leading to the consumption of large amounts of natural resources and, inevitably, the mass discharge of waste. For example, with an increase in the urban population during the process of urbanization, urban residents’ demand for the quantity, quality, and diversity of agricultural products increases. In addition, the large-scale development and population agglomeration, industry, and commerce and service industries increase the demand for ESs and consumption of various resources and energy sources [46]. The advancement of urbanization has also intensified the demand for cultural services among urban residents. Living in a man-made environment formed by steel and concrete for a long time exposes residents to more leisure and entertainment sources, aesthetic functions, and cultural and spiritual services [47]. 

Urbanization will not only affect the S&D of ESs but will also have a profound impact on the relationship between the S&D of ESs [30,35,48]. During the process of urbanization, the disturbance of the ESs supply and the increase in the ESs demand lead to a serious ESs imbalance [21,26]. It is necessary to study the impact of urbanization on the ESs balance [49]. Previous studies focused on describing the characteristics of ESs supply changes caused by land use change in the process of urbanization but neglected to consider the impact of urbanization on the socioecological system as a whole [39,40]. To fill the gap by not only exploring the spatiotemporal pattern of ESs balance and urbanization, but also the relationships between three main urbanization factors, population density, GDP density, and construction land proportion, and ESBI is essential. In addition, the S&D of ESs has a significant scale effect, and there have been few previous studies on the multiple scales associated with the impact of urbanization on the ESs balance. Exploring the impact of urbanization on ESs more precisely can provide references for future ecological management and regional sustainability.

The aim of the construction of the ecological economic belt is to achieve a “win-win” situation between economic development and ecological protection and to establish a compound ecological system with a virtuous cycle of economy, society, and nature. Exploration of the S&D of ESs in the ecological economic belt plays an important role in ecological protection. Ecological progress is a fundamental plan for the sustainable development of the Chinese nation and a basic strategy for the harmonious coexistence of man and nature in the new era. As the largest tributary of the Yangtze River, the Han River ecological Economic Belt (HREEB) is an important part of the Yangtze River Economic Belt. The management and regulation of ESs in the Han River Ecological Economic Belt is an important foundation for the construction of ecological civilization in the Yangtze River Economic Belt. A systematic study of the impact of urbanization on the ESBI in the HREEB can provide a scientific basis for the construction of ecological civilization and the high-quality, sustainable development of the HREEB. Based on this, this study had three research objectives: (1) to evaluate the spatiotemporal distribution characteristics of the ESBI in the HREEB; (2) to reveal the spatial autocorrelation in the ESBI in the HREEB; (3) to identify the multi-scale spatial non-stationarity characteristics of the impact of urbanization elements on the ESBI in the HREEB.

## 2. Materials and Methods

### 2.1. Study Area

**Figure 1:**
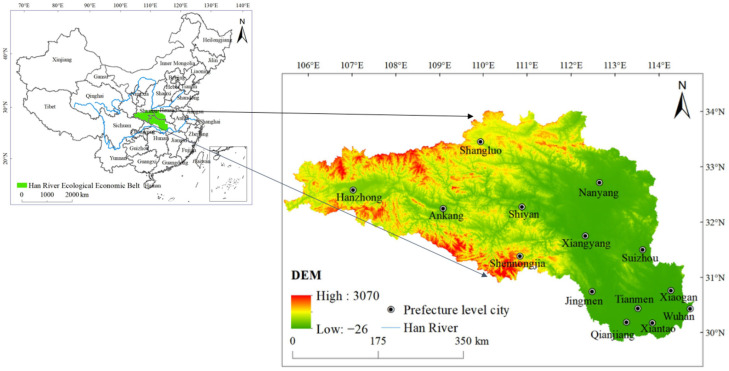
Location of HREEB in China.

The Han River is the largest tributary of the Yangtze River in China. HREEB is an important axis that connects the Wuhan city circle and the western Hubei ecological culture tourism circle (Figure 1). It is an important link between northwest Hubei and Jianghan Plain. HREEB has the functions of “integrating the two circles of Hubei, connecting the belt, connecting the north and the south, connecting the east and the west of China”, and it has an important strategic position and a prominent driving role in the socioeconomic development pattern of Hubei Province. The terrain of HREEB is mainly mountainous (52%), and hilly and plain areas account for approximately 20% of the total area. The special geographical location and topographic conditions make this area “Qinba Wanbao Mountain”, “the cradle of Chinese medicinal materials”, “a natural biological fund bank”, an important station for inland migratory birds, and a potential distribution area for a variety of rare animals and plants. In addition, HREEB is located in a transition zone between subtropical and warm temperate climates and mountains and plains with a complex climate and biological diversity. In recent decades, the rapid advancement of urbanization in the HREEB has intensified the disturbance to the ecosystem, reduced the supply capacity of ESs, and led to a sharp increase in the demand for ESs, resulting in an obvious imbalance between the S&D of ESs. Therefore, it is of great significance to explore the impact of urbanization elements on the ESBI in the HREEB.

### 2.2. Data Sources and Processing

The land use change datasets of 2000 and 2020 used in this study were downloaded from the Data Center of Resources and Environment Science, Chinese Academy of Sciences (RESDC) (http://www.resdc.cn accessed on 1 January 2022), with a 30 m spatial resolution. The spatial distribution datasets of China’s 1 km resolution GDP raster data, DEM, and precipitation data in 2000 and 2020 were also acquired from the RESDC (http://www.resdc.cn accessed on 1 January 2022). Information on the construction land area was extracted from the land use change data set. Data on the intensity of land use were calculated based on the land use change data set. For more details on the calculation process, refer to ref. [50]. Population density datasets in 2000 and 2020 were sourced from Worldpop (https://www.worldpop.org/ accessed on 1 January 2022) with a 100 m spatial resolution. Research on ESs has been explored at multiple scales. Based on previous studies and the size of the study area, we used grids of 5 km and 10 km to explore the impact of urbanization elements on the ESBI so as to break the limitations of traditional administrative region research [5,6,26,51,52].

### 2.3. ESs Balance Index Assessment

This study referred to the ESs matrix method proposed by Burkhard et al. to measure the balance between the S&D of ESs [34,53]. ESs were divided into 23 sub-categories and 3 major categories, including regulating, supplying, and cultural services, and given a score of 0–5 based on the expert scoring results [21,33,34,53], where 0 indicates that there is no relevant supply capacity or demand and 5 indicates that the relevant supply capacity or demand is at the highest level. For more details on the ESs matrix used in this study, refer to refs. [21,33]. The ESs supply index (ESSI), ESs demand index (ESDI), and ESBI were therefore calculated according to the ESs S&D balance matrix. The equations used were as follows:

(1) $$ESSI_{t}=\sum_{j=1}^{m}\sum_{i=1}^{n}\left(LUA_{i,t}\times SM_{ij,t}\right)/\sum_{i=1}^{n}LUA_{i,t},$$

(2) $$ESDI_{t}=\sum_{j=1}^{m}\sum_{i=1}^{n}\left(LUA_{i,t}\times DM_{ij,t}\right)/\sum_{i=1}^{n}LUA_{i,t},$$

(3) $$ESBI_{t}=\sum_{j=1}^{m}\sum_{i=1}^{n}\left(LUA_{i,t}\times BM_{ij,t}\right)/\sum_{i=1}^{n}LUA_{i,t},$$

where *SM_ij,t_*, *DM_ij,t_*, and *BM_ij,t_* represent the supply, demand, and balance matrix of the *j*-th ESs of the *i*-th land use type at time *t*. *LUA_i,t_* is the area of the *i*-th land use type, *n* is the number of land use types, and *m* is the number of ESs types.

### 2.4. Spatial Autocorrelation Analysis

An exploratory spatial data analysis method was used to test whether there were significant spatial autocorrelation relationships between urbanization and the ESBI [5,50,54]. In this study, global bivariate spatial autocorrelation and local bivariate spatial autocorrelation were used to measure the spatial correlation. The global spatial autocorrelation is usually measured by Moran’s *I* index, which can reflect the similarities between regional unit attribute values of spatial adjacency, while the local bivariate Moran’s *I* is used to test whether there are spatial correlations between different units. The calculation equations are shown in Equations (4) and (5):

(4) $$I_{eu}=\frac{N\sum_{i}^{N}\sum_{j\neq i}^{N}W_{ij}z_{i}^{e}z_{j}^{u}}{(N-1)\sum_{i}^{N}\sum_{j\neq i}^{N}W_{ij}},$$

(5) $$I^{\prime }{}_{eu}=z^{e}\sum_{j=1}^{N}W_{ij}z_{ij}^{u},$$

where *I_eu_* and *I^′^_eu_* represent the global Moran’s bivariate *I* and the local bivariate Moran’s *I* of the ESBI and urbanization elements, respectively. *W_ij_* is the spatial weight matrix. *z_i_^e^* and *z_j_^e^* are the values of standardization of the ESBI and urbanization elements, respectively. According to the local spatial autocorrelation results, there are four types of calculation results: high–high, low–low, high–low, and low–high.

### 2.5. Geographically Weighted Regression Model

In this study, the geographically weighted regression (GWR) model was used to measure the spatial non-stationary characteristics of the impact of urbanization elements on the ESBI in the HREEB [55,56,57]. The ordinary least squares (OLS) model implies the assumption of spatial homogeneity. The GWR is a spatial regression model that is based on the idea of local smoothing. It can not only effectively estimate data through spatial autocorrelation but can also reflect the spatial heterogeneity of parameters in different regions [58,59,60]. The “adaptive” and modified Akeck information criterion (AICc) was selected for the GWR analysis. In the GWR results, the corrected R^2^ reflects the explanatory power of independent variables and was used to test the model performance. The calculation equation was as follows:

(6) $$y_{i}=\beta_{0}\left(u_{i},v_{i}\right)+\sum_{k=1}^{p}\beta_{k}\left(u_{i},v_{i}\right)x_{ik}+\varepsilon_{i}$$

where *y_i_* is the ESBI value of sampling unit *i*. *(u_i_,v_i_)* is the constant term of the sampling unit. *β_k_(u_i_,v_i_)* is the coefficient of the *k*-th independent variable of the sampling unit, *x_ik_* is the *k*-th independent variable of the sampling unit *I*, *β_0_* is the intercept, and *ε_i_* is the random error. 

This study investigated the urbanization level of the HREEB from three urbanization elements: population urbanization, economic urbanization, and spatial urbanization. We chose population density to represent population urbanization, GDP density to represent economic urbanization, and the proportion of construction land to represent spatial urbanization [5,7,31]. In addition, physical and socioeconomic factors were selected as control variables. Specifically, DEM represents the topographic characteristics of the study area, precipitation represents climate factors, and land use intensity represents the intensity of human activities [33,43].

## 3. Results

### 3.1. Spatiotemporal Patterns of ESs in the HREEB

**Figure 2:**
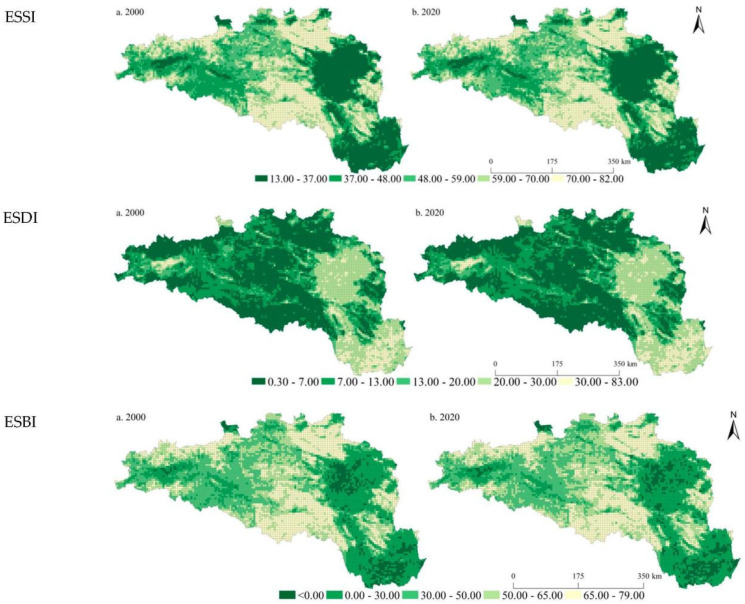
Spatial pattern of the ESSI, ESDI, and ESBI at the 5 km grid scale in the HREEB in China.

**Figure 3:**
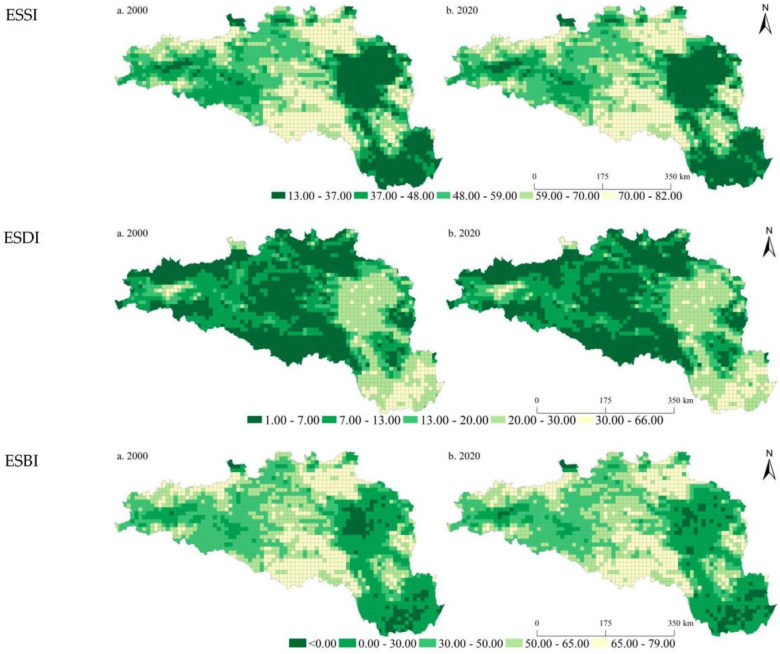
Spatial pattern of the ESSI, ESDI, and ESBI at the 10 km grid scale in the HREEB in China.

We mapped the spatiotemporal patterns of the ESSI, ESDI, and ESBI of the HREEB at 5 km and 10 km grid scales in 2000 and 2020 (Figure 2 and Figure 3). In 2000 and 2020, the ESSIs of the HREEB were 53.02 and 53.04, the ESDIs were 12.64 and 12.86, and the ESBIs were 40.38 and 40.18, respectively. It was found that the ESSI and ESDI increased slightly in the HREEB during the study period, and the ESBI showed a decreasing trend. During the study period, the proportions of units with declining trends in the ESSI, ESDI, and ESBI at the 5 km scale were 57.04%, 39.47%, and 56.22%, respectively. At the 10 km scale, the proportional decreases in the ESSI, ESDI, and ESBI were 57.15%, 35.96%, and 57.67%, respectively. Spatially, we found that the ESSI was evidently lower in the middle and lower reaches of the Han River than that in the upper reaches of the Danjiangkou reservoir. The ESSI was particularly low in the Wuhan city circle, Xiangyang, and Nanyang. On the whole, it was found that the ESSI was higher in high-altitude areas than in low-altitude areas, and the ESDI was evidently higher in low-altitude areas than in high-altitude areas. Areas with an ESBI of less than 0 increased from 2000 to 2020, mainly in the Wuhan city circle and in Xiangyang and Nanyang. During the study period, units with evident increases in the ESSI were mainly distributed in mountainous areas with higher altitudes, while the ESDI increased significantly in plain areas with lower altitudes. Similarly, the ESBI decreased significantly in low-altitude areas. By comparing the distribution results of ESs at different scales, the spatial distribution characteristics of the ESSI, ESDI, and ESBI at 5 km and 10 km scales were found to be consistent, but a more detailed scale could provide more detailed results.

### 3.2. Spatiotemporal Patterns of the Urbanization Level in the HREEB

**Figure 4:**
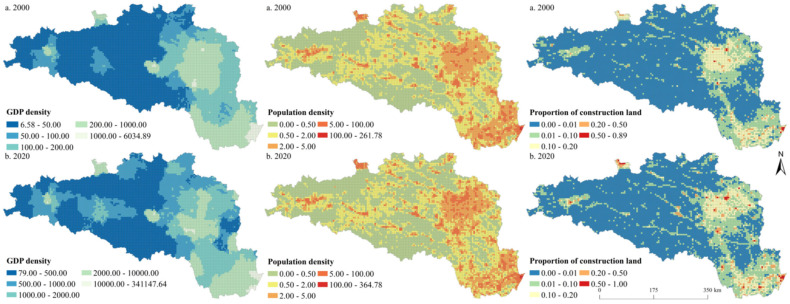
Spatial pattern of urbanization elements at the 5 km grid scale in the HREEB in China.

**Figure 5:**
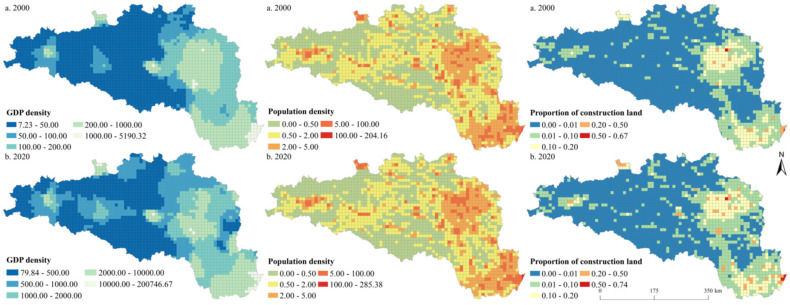
Spatial pattern of urbanization elements at the 10 km grid scale in the HREEB in China.

We drew the spatial pattern of urbanization elements of the HREEB at 5 km and 10 km grid scales in 2000 and 2020 (Figure 4 and Figure 5). During the study period, the economic development level of the HREEB evidently improved, and the economic development level of the middle and lower reaches of the Han River became higher than that of the Danjiangkou Reservoir area and the upper reaches of the Han River. The regions with a high GDP density were mainly distributed in the core areas of prefecture-level cities, especially Wuhan, Xiangyang, Nanyang, Hanzhong, and Shangluo. Similarly, the population density of HREEB was significantly higher in plain areas than in mountainous areas, and the economic development level of the middle and lower reaches of the HREEB was significantly higher than that of the Danjiangkou Reservoir area and the upper reaches of the HREEB. The population density was obviously higher in the core areas of prefecture-level cities and areas along the main traffic routes. The proportion of construction land was similar to the GDP density and population density in terms of spatial distribution. On the whole, it was found that the urbanization level of the HREEB evidently improved during the study period, and the economic development level of the middle and lower reaches of HREEB was significantly higher than that of the Danjiangkou Reservoir area and the upper reaches of the HREEB. The urbanization level in the core areas of prefecture-level cities and areas along major traffic routes (e.g., the Erlianhot–Guangzhou Expressway, the Wuhan–Xi’an High-speed Railway, and the Shanghai–Shaanxi Expressway) was higher.

### 3.3. Bivariate Spatial Autocorrelation Analysis

**Figure 6:**
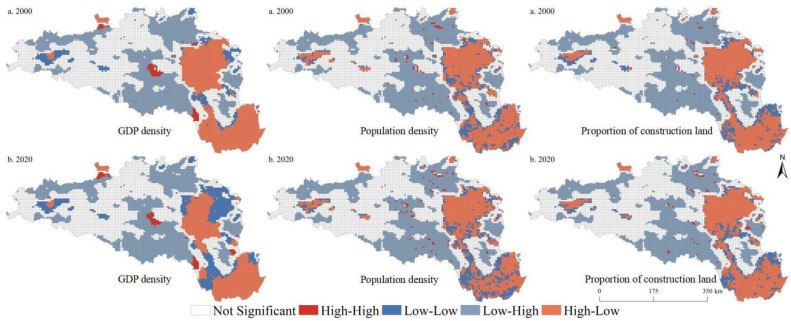
LISA map of urbanization elements and ESBI at the 5 km grid scale in the HREEB in China.

**Figure 7:**
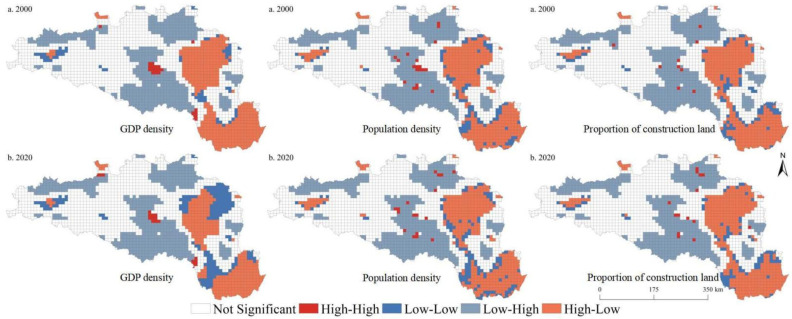
LISA map of urbanization elements and the ESBI at the 10 km grid scale in the HREEB in China.

In 2000, the global bivariate spatial autocorrelation indexes between the GDP density, population density, and proportion of construction land and ESBI were −0.59, −0.30, and −0.44, respectively. In 2020, the global bivariate spatial autocorrelation indexes between the GDP density, population density, and the proportion of construction land and ESBI were, respectively, −0.57, −0.28, and −0.21 at the 5 km grid scale. Similarly, we found that, in 2000, the global bivariate spatial autocorrelation indexes between the GDP density, population density, and proportion of construction land and ESBI were −0.61, −0.27, and −0.45, respectively. In 2020, the global bivariate spatial autocorrelation indexes between the GDP density, population density, and the proportion of construction land and ESBI were, respectively, −0.59, −0.25, and −0.22 at the 10 km grid scale. Significant spatial autocorrelation relationships were identified between the ESBI and urbanization elements. Figure 6 and Figure 7 show the LISA map between urbanization elements and the ESBI. Overall, it was found that the spatial distribution pattern characteristics of urbanization factors and the ESBI were generally consistent. Specifically, high–low (high urbanization level–low ESBI) and low–high (low urbanization level–high ESBI) types were the main relationship types identified between urbanization elements and the ESBI in the HREEB. The high–low type was mainly distributed in the middle and lower reaches of the Han River, while the low–high relationship type was mainly distributed in the Danjiangkou Reservoir and the upper reaches of the Han River. In addition, some low–low types and high–high types were dispersed in the areas surrounding high–low and low–high relationship types.

### 3.4. Spatially Varying Relationships

**Figure 8:**
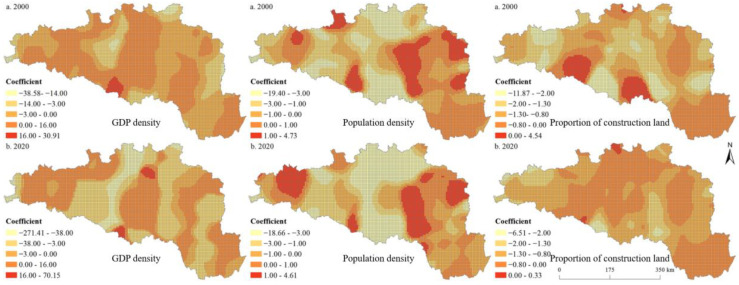
Spatial distribution of the regression coefficient of the urbanization elements at the 5 km grid scale in the HREEB in China.

**Figure 9:**
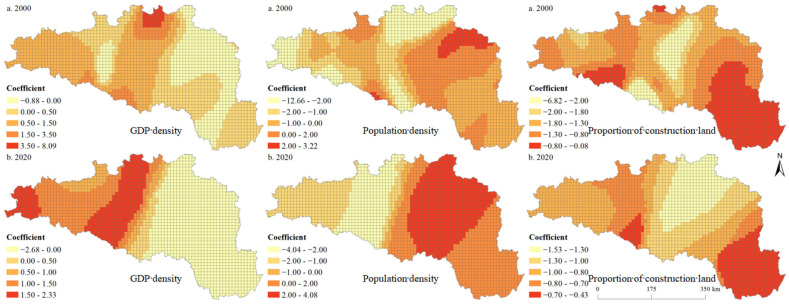
Spatial distribution of the regression coefficient of urbanization elements at the 10 km grid scale in the HREEB in China.

Table 1 and Table 2 present the results of the OLS model at the 5 km and 10 km grid scales, respectively. The variance inflation factor was less than 4 for all variables, indicating that there was no multicollinearity among variables, and the model was reasonably set. The model results showed that the model could explain the impact of urbanization factors on ESBI well. Most of the influencing factors reached a significant level. We found that the GWR model significantly outperformed the OLS model (Table 3). Table 4 and Table 5 show the statistical analysis results of the fitting coefficient values of the sample points. Figure 8 and Figure 9 present the regression coefficient of the urbanization elements at the 5 km and 10 km grid scales in the HREEB. The results show that the effects of urbanization elements on ESBI were spatially and temporally heterogeneous, with significant scale effects.

Specifically, the proportion of construction land was mainly negatively correlated with ESBI, while population density was positively correlated with ESBI. There were both positive and negative correlations between GDP density and ESBI (Table 2 and Table 3). In addition, there was also a significant positive correlation between elevation and ESBI at the 5 km and 10 km grid scale. Land use intensity and precipitation were negatively correlated with ESBI in 2000, while they were positively correlated with ESBI in 2020 at the 5 km grid scale. Land use intensity was negatively correlated with ESBI at the 10 km grid scale during the study period. As shown in Table 3, the fitting coefficients R^2^ of the 2000 and 2020 models were 0.89 and 0.88, respectively, at the 5 km grid scale, and the adjusted fitting R^2^ coefficients were 0.91 and 0.86, respectively, at the 10 km grid scale, indicating that the GWR model can better model the impact of urbanization elements on the ESBI. The results of the GWR model showed that the influence of the GDP density on the ESBI was either positive or negative, indicating that the influence of the GDP density on the ESBI was unstable and had the characteristics of non-stationarity in geographical space. The positive regression coefficient at the 10 km grid scale was evidently higher than that at the 5 km grid scale and was mainly distributed in the Danjiangkou reservoir area and the upstream area. Similarly, the impact of population density on the ESBI can be either positive or negative. Specifically, at the scales of the 5 km and 10 km grids, the areas where population density had a positive impact on ESBI were mainly distributed in the middle and lower reaches of the HREEB. At the scale of the 10 km grid, the proportion of construction land had a negative impact on the ESBI, while at the scale of the 5 km grid, the proportion of construction land had a positive impact on the ESBI only in a small proportion of units, and the overall impact was negative.

## 4. Discussion

### 4.1. Impact of Urbanization Elements on ESs Balance Index

The supply of ESs reflects the ability of an ecosystem to produce ecosystem products and services that can be used by human beings, while the demand for ESs reflects the demand intensity of human beings to use and consume ecosystem products and services [33]. As one of the most influential human activities, urbanization will seriously interfere with the ESs supply. In addition, the promotion of urbanization will increase the demand level for ESs, and the development of population urbanization and economic urbanization will lead to increases in resource consumption and pollutant emissions, thereby increasing the demand for ESs [26,61]. Therefore, there was a close relationship between urbanization and ESBI. Based on bivariate spatial autocorrelation and GWR models, this study analyzed the spatial relationship between urbanization and ESBI at 5 km and 10 km scales, which was essential to achieve the sustainable management of urban ecosystems.

The global bivariate spatial autocorrelation results showed that there was a negative correlation between urbanization factors and the ESBI at the 5 km and 10 km scales. The high–low (high urbanization level–low ESBI) and low–high (low urbanization level–high ESBI) types were the main relationship types. On the whole, the GWR model showed that urbanization factors had a significant negative impact on the ESBI, but there was significant spatial heterogeneity. Specifically, the influence of the GDP density and population density on the ESBI was either positive or negative, indicating that the influence of them on the ESBI was unstable and had the characteristics of non-stationarity in geographical space. At the same time, the overall impact of the proportion of construction land was negative. Previous studies have also evidenced similar findings [30]. For example, Deng et al. (2021) provided evidence that urbanization offsets the improvement in ESs brought about by ecological construction [61]. In addition, the dislocation of urbanization and ecological construction aggravates the decline in the ESBI [29]. Lapointe (2020) reported that both rural and urban residents feel negative impacts from ecosystem disservices, urbanization would reduce the relationship between people and nature, and urban residents were less satisfied with the services provided by ecosystems than rural residents in the process of urbanization [45]. Specifically, urban expansion would affect the material and energy flow, water cycle, carbon cycle, and other ecological processes by squeezing out other land types and limiting the supply of ESs [62,63,64,65]. The increase in the urban population and the development of economic urbanization require more production space and living space, which would inevitably further squeeze out the ecological space and reduce the supply capacity of ESs [66,67]. In addition, some scholars found that there was a “positive correlation” or “inverted U” relationship between urbanization elements and ESs [5]. For example, Chen et al. (2022) found a significant *U*-shaped relationship between urbanization and ESs and a significant scale effect of urbanization on ESs. In other words, in the early stage of urbanization, the promotion of urbanization leads to serious degradation of ecosystem functions, but when urbanization reaches a certain level, it promotes the improvement of ESs. In addition, some scholars have also found that the degree of coupling and coordination between the urbanization system and the ecosystem increased on a municipal scale in HREEB [68].

### 4.2. Policy Implications

The rapid development of urbanization in the HREEB has led to an evident decrease in the ESSI and an obvious increase in the ESDI in some units. Overall, the ESBI showed a decreasing trend. However, overall, the total supply of ESs can still satisfy the total demand. Despite this, there was a large mismatch between S&D in space. In particular, in the middle and lower reaches of the Han River and the surrounding areas of prefectural cities, the S&D deficit of ESs was serious. To scientifically measure the region of profit and loss of the S&D of ESs in the HREEB and reveal the spatial non-stationarity of the impact of urbanization on the ESBI, the balance between the S&D of ESs between regions can be balanced as a key factor in ecological management and the gradual deficit trend of the ESBI can be curbed with urbanization development. Based on our findings, we proposed the following possible suggestions.

(1) Based on the results for the S&D of ESs and the balance between them, the deficit and surplus areas of ESs can be identified. Different ecological function zones can be defined according to the S&D of ESs, and the dominant functions can be clearly defined. The balance between the S&D of ESs in HREEB can be achieved by complementing the regional functions. This process can achieve a balance between the S&D of ESs through the spatial flow of ESs. In addition, the balance of regional ESs can be achieved by increasing the supply of ESs or reducing the demand for ESs. For the key urbanization region, the demand capacity of ESs was strong, but the supply capacity of ESs was weak. Therefore, the deficit of ESs in the key urbanization region needs to be balanced in regard to the whole HREEB region or a larger region [39]. The boundary restrictions of administrative regions need to be broken, and collaborative governance of ecosystems among regions needs to be achieved [33].
(2) According to the spatial non-stationarity characteristics of the impact of urbanization on the ESBI, suggestions can be made to alleviate the trade-off between them. In areas where urbanization had a serious impact on the ESBI, full attention should be paid to alleviating population and economic pressure, and the red line of arable land and ecological land should be adhered to, ensuring the balance between the grain S&D was achieved, and the demand for ecological land was met. It is suggested that the ecological corridors between surplus and deficit regions of the ESs S&D should be optimized, and a regional ecological security pattern should be built in the HREEB [39,69].
(3) In addition, cooperation between regions within the HREEB should be strengthened to build a perfect ecological compensation mechanism. The ecological compensation mechanism can guarantee the source of funds for ecological protection, and it can also force the transformation and upgrading of industries [70,71,72]. At present, the compensation mechanism has not been fully implemented in the Han River basin, and the ecological compensation standards in the upstream and downstream transboundary areas are unclear. The Hubei Provincial Government has issued relevant documents to support local governments in the Han River Basin to establish a horizontal compensation mechanism for ecological protection and increase support for transfer payments to key ecological function zones, major agricultural production areas, and poverty-stricken areas.
(4) At present, the contradiction between accelerating economic growth and strengthening ecological protection is prominent in the HREEB. HREEB ecological protection can be based on the different functions of the upper, middle, and lower reaches of the region and the different geographical conditions and climatic disturbance, combined with the characteristics of the ESs S&D. In terms of agricultural development, it was suggested that a modern agricultural operation system should be built, the support for agricultural modernization should be strengthened, eco-friendly agriculture should be vigorously developed, the construction of modern agricultural demonstration zones should be accelerated, and our ability to ensure food security should be enhanced.

### 4.3. Limitations and Prospects

This study revealed the non-stationary patterns of the impact of urbanization elements on the ESBI in the HREEB region at multiple scales, providing a new perspective for understanding the interference of the urbanization process on the ESBI. However, there were still some deficiencies in this study, as follows: (1) although the ESs matrix method provides a pathway for the measurement of the ESBI, it still has a degree of subjectivity, so it needs to be further improved in future research to improve the assessment accuracy of the ESBI. (2) This study used the GWR model to analyze the influences of urbanization factors on the ESBI at the grid scale, but the measurement indexes for the urbanization level and selection of influencing factors are still insufficient. Because the formation mechanism in the S&D balance of ecological systems was complex, not only due to natural factors such as the topography, climate, and hydrology, and human factors, such as society and economy, the behavior of individual factors, such as the functions between various factors also leads to ESs changes combined with sudden natural disasters and human activities over long periods of time. At present, there is no clear explanation for the mechanism influencing the S&D balance of ESs. In the future, similar studies exploring other scales and regions should further improve the urbanization evaluation system and the selection of control variables.

## 5. Conclusions

This study measured the spatiotemporal patterns of the ESSI, ESDI, and ESBI in the HREEB with the ESs matrix method using land use change data. A bivariate spatial autocorrelation model was used to verify the spatial agglomeration characteristics between urbanization and the ESBI. Then, the GWR was used to measure the effects of multi-scale spatially varying relationships in urbanization on the ESs balance in the HREEB. The findings of this study were as follows:

(1) During the study period, the ESSI and ESDI of the HREEB increased, while the ESBI showed a decreasing trend. Overall, we found that the ESSI and ESBI were significantly higher in mountain areas than in plain areas, and on the contrary, the ESDI was higher in plain areas than in mountain areas.
(2) The urbanization level of the HREEB improved during the study period. Generally, we found that the urbanization level in the middle and lower reaches of the Han River was higher than that in the Danjiangkou Reservoir area and the upper reaches of the Han River.
(3) There was a significant spatial autocorrelation between urbanization and the ESBI. High–low (high urbanization level–low ESBI) and low–high (low urbanization level–high ESBI) types were the main relationship types identified between urbanization elements and the ESBI in the HREEB during the study period.
(4) The GWR model can model the multi-scale spatial relationship between urbanization factors and the ESBI well. We found that the impact of urbanization factors on the ESBI can be positive or negative, but it varies significantly in space.

## Figures and Tables

**Table 1:** Result of the OLS model at the 5 km grid scale.

Variable	2000				2020			
	Coefficient	Standardized Coefficients	Sig.	VIF	Coefficient	Standardized Coefficients	Sig.	VIF
Intercept	0.691	0.004	0.000		0.649	0.004	0.000	
Proportion of construction land	−1.588	0.029	0.000	2.125	−1.243	0.021	0.000	1.915
Population density	0.856	0.079	0.000	2.004	0.710	0.100	0.000	3.071
GDP density	−0.020	0.037	0.730	1.973	−0.091	0.074	0.000	2.262
Elevation	0.353	0.007	0.000	1.366	0.396	0.007	0.000	1.636
Land use intensity	−0.016	0.005	0.000	1.108	0.001	0.004	0.707	1.097
Precipitation	−0.018	0.008	0.012	1.171	0.093	0.006	0.000	3.070

**Table 2:** Result of the OLS model at 10 km grid scale.

Variable	2000				2020			
	Coefficient	Standardized Coefficients	Sig.	VIF	Coefficient	Standardized Coefficients	Sig.	VIF
Intercept	0.763	0.010	0.000		0.731	0.010	0.000	
Proportion of construction land	−1.403	0.053	0.000	2.486	−1.034	0.035	0.000	2.119
Population density	0.701	0.074	0.000	2.337	0.594	0.141	0.003	3.012
GDP density	0.053	0.073	0.527	2.630	−0.081	0.083	0.395	2.470
Elevation	0.309	0.013	0.000	1.458	0.307	0.013	0.000	1.958
Land use intensity	−0.298	0.014	0.000	1.380	−0.257	0.013	0.000	1.425
Precipitation	−0.090	0.014	0.000	1.175	0.036	0.011	0.000	1.612

**Table 3:** Test result of the GWR model.

Scale/Year	2000		2020	
5 km grid scale	Variable name	Variable	Variable name	Variable
	Bandwidth	35,386.88	Bandwidth	35,386.88
	Residual Squares	12.44	Residual Squares	9.37
	Effective Number	176.98	Effective Number	197.12
	Sigma	0.06	Sigma	0.05
	AICc	−11,394.77	AICc	−13,977.34
	R^2^	0.89	R^2^	0.88
	R^2^Adjusted	0.89	R^2^Adjusted	0.87
10 km grid scale	Bandwidth	68,965.89	Bandwidth	157,468.11
	Residual Squares	6.67	Residual Squares	10.34
	Effective Number	95.50	Effective Number	35.95
	Sigma	0.06	Sigma	0.07
	AICc	−4669.99	AICc	−5116.64
	R^2^	0.92	R^2^	0.86
	R^2^Adjusted	0.91	R^2^Adjusted	0.86

**Table 4:** Statistical results of the GWR model coefficient at the 5 km scale.

Variable	2000								2020							
	Min	Lower Quantile	Median	Upper Quantile	Max	Mean	Negative Ratio	Positive Ratio	Min	Lower Quantile	Median	Upper Quantile	Max	Mean	Negative Ratio	Positive Ratio
Proportion of construction land	−11.87	−1.56	−1.06	−0.65	4.54	−1.20	0.93	0.07	−6.50	−1.08	−0.80	−0.58	0.33	−0.88	0.99	0.01
Population density	−19.40	−2.21	−0.35	0.39	4.72	−1.23	0.58	0.42	−18.66	−3.10	−0.51	0.41	4.61	−1.53	0.65	0.35
GDP density	−38.57	−1.01	−0.02	0.51	30.90	−0.40	0.51	0.49	−271.41	−9.82	−0.64	1.28	70.15	−9.17	0.57	0.43
Elevation	−9.74	0.34	0.58	1.87	5.19	1.16	0.05	0.95	−8.35	0.28	0.51	1.64	4.91	1.03	0.04	0.96
Land use intensity	−1.55	−0.02	0.00	0.00	0.04	−0.09	0.68	0.32	−1.50	−0.02	0.00	0.00	0.03	−0.09	0.65	0.35
Precipitation	−1.62	−0.22	0.03	0.33	1.39	0.06	0.47	0.53	−1.96	−0.10	0.11	0.41	5.61	0.16	0.34	0.66

**Table 5:** Statistical results of the GWR model coefficient at the 10 km scale.

Variable	2000								2020							
	Min	Lower Quantile	Median	Upper Quantile	Max	Mean	Negative Ratio	Positive Ratio	Min	Lower Quantile	Median	Upper Quantile	Max	Mean	Negative Ratio	Positive Ratio
Proportion of construction land	−6.82	−1.60	−1.22	−0.75	−0.08	−1.25	1.00	0.00	−1.53	−1.17	−0.91	−0.75	−0.42	−0.96	1.00	0.00
Population density	−12.66	−1.55	−0.22	0.27	3.21	−0.81	0.67	0.33	−4.03	−1.81	0.37	2.24	4.08	0.31	0.39	0.61
GDP density	−0.87	−0.02	0.22	0.90	8.08	0.55	0.27	0.73	−2.67	−0.37	−0.05	1.32	2.32	0.29	0.51	0.49
Elevation	−0.20	0.31	0.58	1.10	4.19	0.92	0.04	0.96	0.09	0.26	0.40	0.56	1.55	0.44	0.00	1.00
Land use intensity	−0.89	−0.41	−0.18	−0.06	0.05	−0.26	0.99	0.01	−0.66	−0.47	−0.20	−0.10	−0.06	−0.28	1.00	0.00
Precipitation	−1.38	−0.19	0.00	0.14	0.81	−0.04	0.50	0.50	−0.32	−0.08	0.03	0.16	0.39	0.04	0.45	0.55

## Data Availability

Not applicable.
